# Supplementary material for: Tannic acid shaped microbiome composition in midguts and rearing microcosms of Aedes triseriatus (Say)
Source: Front Microbiol. 2026 Jan 26;17:1755894. doi: 10.3389/fmicb.2026.1755894 (PMC12884536; doi:10.3389/fmicb.2026.1755894)
Supplement: Supplementary file 1 [file Data_Sheet_1.pdf]

Supplemental Material

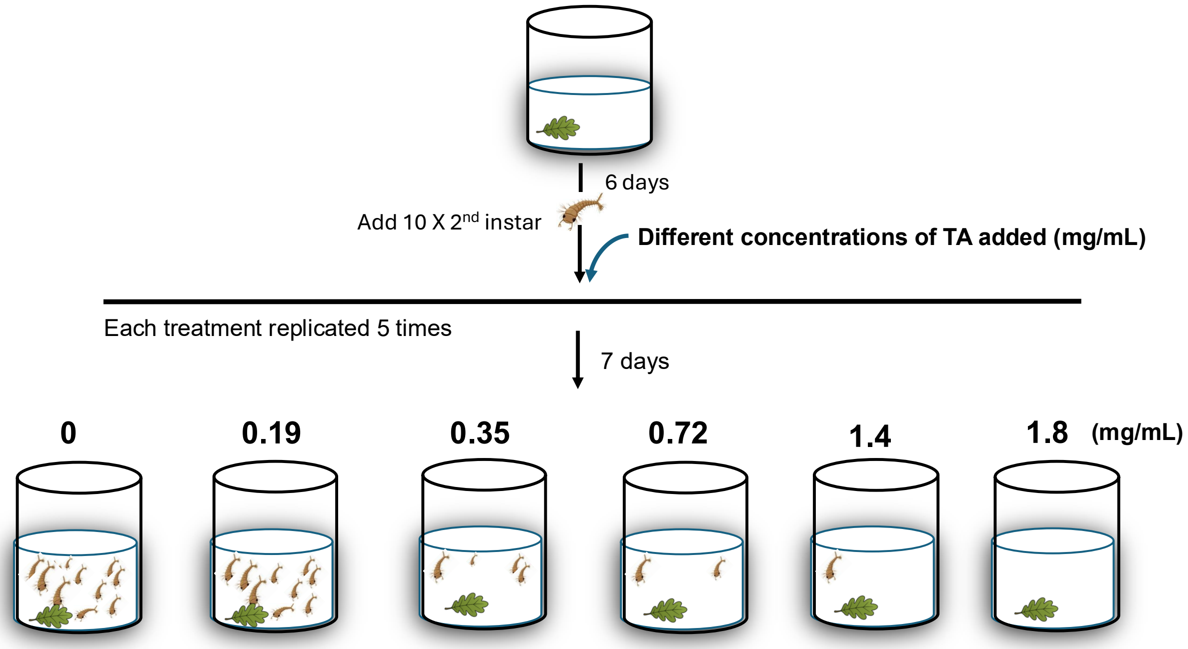

Supplementary Figure 1. Schematic of the experimental design examining the effects of TA on mosquito larval survival. Microcosms consisted of 1 g of beech leaf litter, 80 mL of Milli-Q water, and 1 mL of microbial inoculum. Following a 6-day incubation period, ten second-instar larvae were introduced into each microcosm along with supplemented TA at concentrations of 0.00, 0.19, 0.35, 0.72, 1.4, and 1.8 mg/mL. Larval survivorship was assessed after one week of treatment. Five replicates were included for each treatment group.

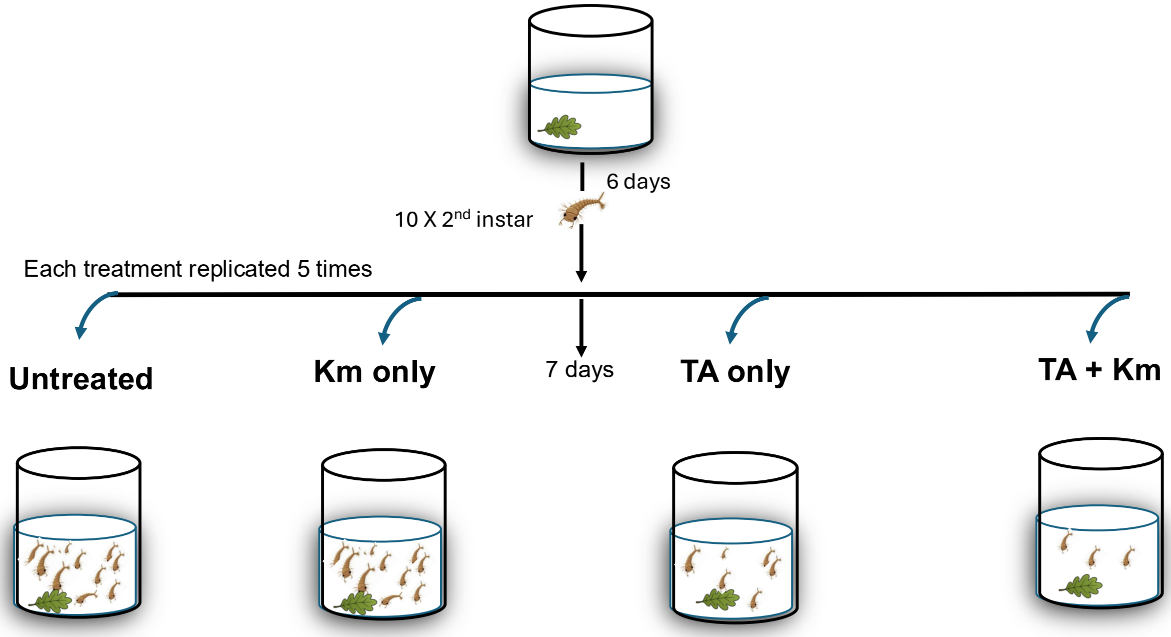

Supplementary Figure 2. Schematic of the experimental design examining the effects of TA and antibiotic treatments on mosquito larval survival. Four treatment groups were established: (1) untreated, (2) Kanamycin-only (100 µg/mL), (3) TA-only (sublethal dose, 0.35 mg/mL), (4) combined TA + kanamycin (0.35 mg/mL TA + 100 µg/mL kanamycin). Microcosms were added either 2 mL of TA solution plus 2 mL of Milli-Q water (treatments) or 4 mL of Milli-Q water only (untreated control). Ten second-instar *Ae. triseriatus* larvae were introduced into microcosm at the time of treatment. Larval survival was assessed after seven days of exposure. Five replicates were included for each treatment group.

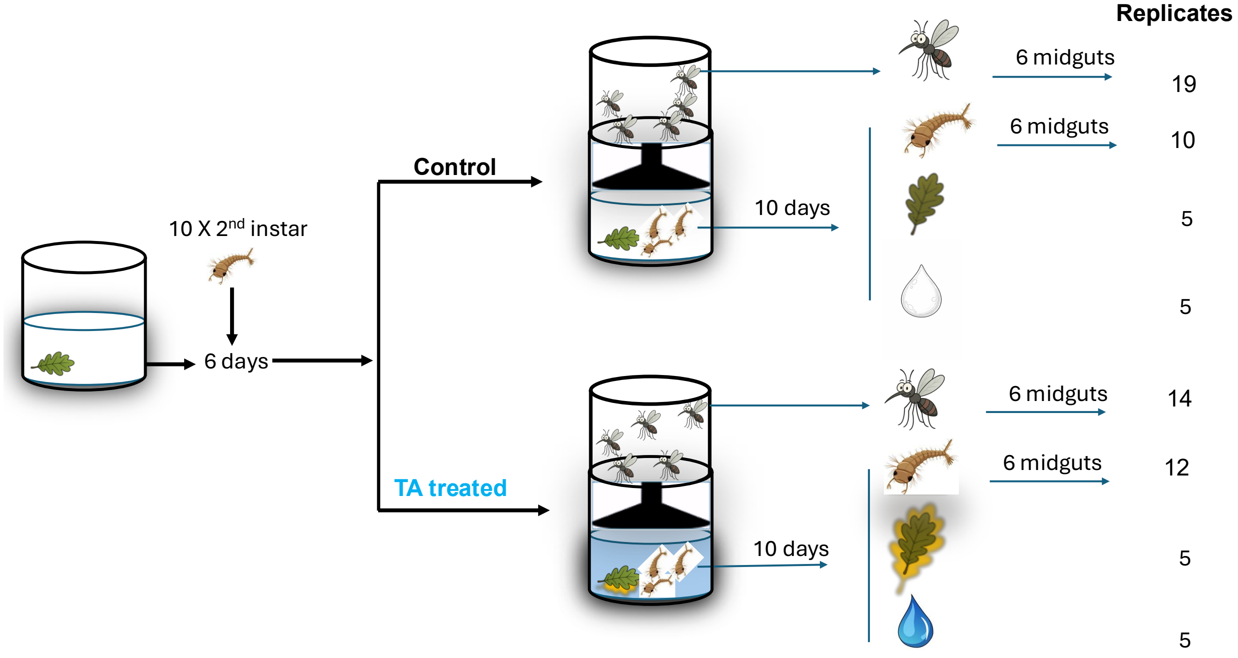

Supplementary Figure 3. Schematic of the experimental design examining the effects of TA on the microbiome of larval mosquitoes, adult mosquitoes, rearing water, and leaf surfaces. Microcosms were established as described above.

For the control treatment, after a 10-day incubation period, fourth-instar larvae were collected, and six midguts were dissected and pooled for microbial DNA extraction (10 replicates). Upon adult emergence, six adults were collected, and six midguts were dissected and pooled for microbial DNA extraction (total 19 replicates). Rearing water samples were collected by centrifugation at 15,000 rpm for 10 min at 4 °C (five replicates). Leaf material, after removal of surface water, was flash-frozen in liquid nitrogen and ground using a sterile pestle (five replicates).

For the TA treatment (0.35 mg mL^{-1}), after a 10-day incubation period, fourth-instar larvae were collected, and six midguts were dissected and pooled for microbial DNA extraction (12 replicates). Following adult emergence, adults were collected, and six midguts were pooled for microbial DNA extraction (14 replicates). Rearing water samples were collected by centrifugation at 15,000 rpm for 10 min at 4 °C (five replicates). Leaf material was processed as described for the control group, with five replicates included.

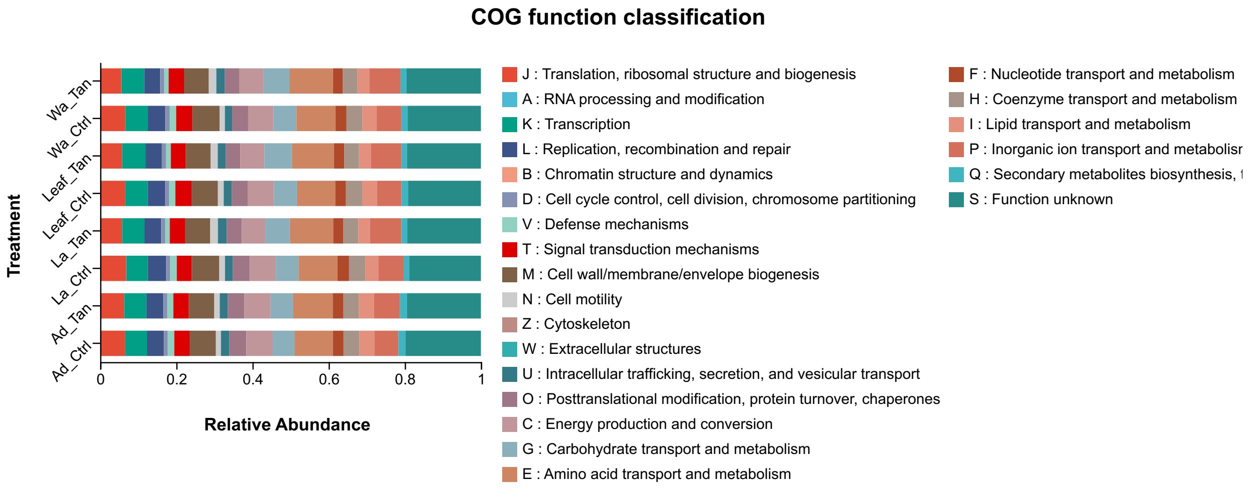

Supplementary Figure 4. COG function classification of the predicted functions by PICRUSt2 analysis. Ad_Ctr, adult mosquitoes without tannic acid treatment; Ad_Tan, adult mosquitoes with tannic acid treatment; La_Ctr, larval mosquitoes without tannic acid treatment; Lar_Tan, larval mosquitoes with tannic acid treatment; Leaf_Ctr, leaf without tannic acid treatment; Leaf_Tan, leaf with tannic acid treatment; Wa_Ctr, water without tannic acid treatment; Wa_Tan, water with tannic acid treatment.

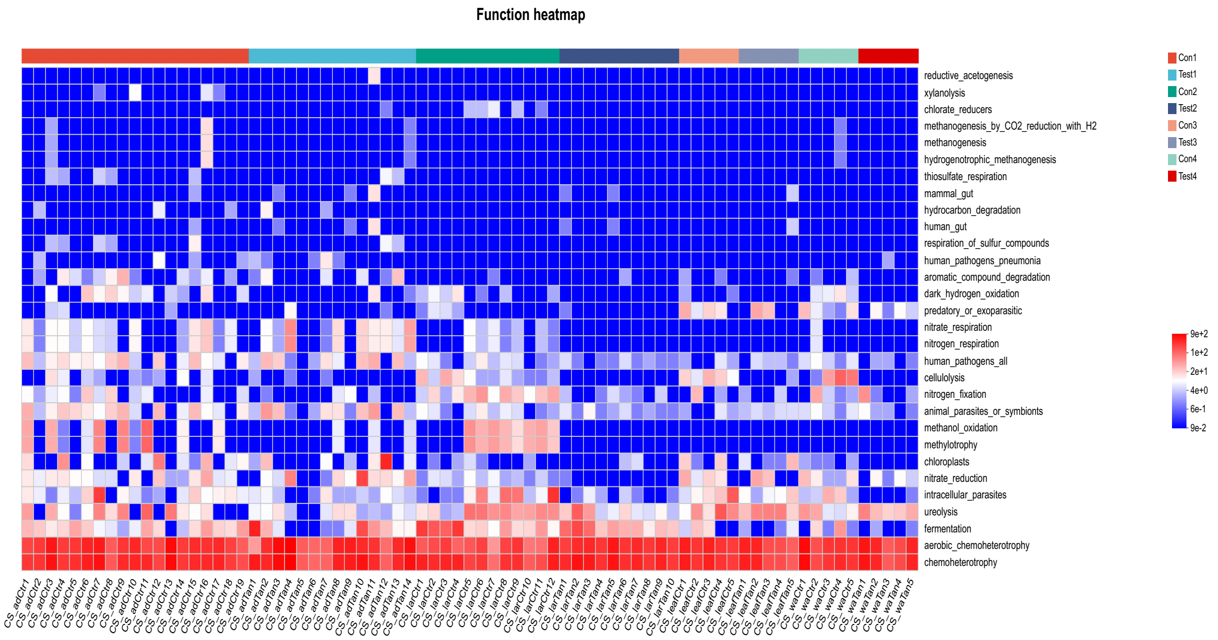

Supplementary Figure 5. Functional prediction among all groups by FAPROTAX.
